# PROTOCOL: Exploring the effect of case management in homelessness per components: A systematic review of effectiveness and implementation, with meta‐analysis and thematic synthesis

**DOI:** 10.1002/cl2.1220

**Published:** 2022-02-23

**Authors:** Alison L. Weightman, Mark J. Kelson, Ian Thomas, Mala K. Mann, Lydia Searchfield, Ben Hannigan, Robin J. Smith, Simone Willis, Rhiannon Cordiner

**Affiliations:** ^1^ Specialist Unit for Review Evidence (SURE) Cardiff University Cardiff UK; ^2^ Alan Turing Institute, School of Mathematics University of Exeter UK; ^3^ Wales Institute of Social and Economic Research and Data (WISERD) Cardiff University Cardiff UK; ^4^ Mental Health Nursing, School of Healthcare Sciences Cardiff University Cardiff UK; ^5^ School of Social Sciences Cardiff University Cardiff UK

## Abstract

This is the protocol for a Campbell review. The objectives are as follows: To carry out a mixed methods review to summarise current evidence relating to the components of case‐management interventions for people experiencing homelessness.

## 1 BACKGROUND

### 1.1 The problem, condition or issue

Adequate housing is a basic human right linked to other core values such as dignity, fairness, equality, respect, and independence (United Nations, 2020). Homelessness, as the ‘lack of minimally adequate housing’ (Busch‐Geertsema et al., 2016), is therefore a human rights issue. However, homelessness is more than the lack of housing as a material resource (Nicholls, 2010; Somerville, 1992). ‘Adequate’ housing is stable, that is, without fear of loss, and provides privacy and personal space for people to conduct their lives as they wish (Amore et al., 2011; Edgar, 2009).

People experiencing homelessness (PEH) can include those living on the street (‘roofless’), people living in temporary or crisis accommodation, such as hostels and shelters (‘houseless’), and people living in accommodation that is either insecure, for example, people about to face eviction or ‘sofa surfers’, or inadequate, for example, severely overcrowded housing. The United Nations (United Nations, 2020) reports that globally, more than 1.8 billion people lack adequate housing, with 150 million people experiencing homeless.

Given homelessness can cover a range of experiences, its causes are varied, often intersecting, and context specific. In the United Kingdom, from which this review originates, longitudinal analysis using national surveys has identified that poverty, particularly during childhood, was a strong predictor of homelessness; labour and housing market pressures can also limit access to and security of housing in adulthood (Fitzpatrick & Johnsen, 2012). In Denmark however, analysis using population‐level data relating to homeless shelters found that shelter use was concentrated amongst people with complex issues—such as drug and alcohol use issues—rather than associated with poverty (Benjaminsen, 2016). Furthermore, the causes of homelessness vary for different social groups within society, for example, LGBTQ + people (Dunne et al., 2002) and families versus lone PEH (Baptista et al., 2017).

PEH have lower life expectancy (Hwang, 2000; Nusselder et al., 2013), increased risk of mortality (Baggett et al., 2013; Fazel et al., 2014; Ivers et al., 2019; Seastres et al., 2020), and a high prevalence of mental health issues including depression and schizophrenia (Ayano et al., 2021; Gutwinksi et al., 2021). Compared to people of similar characteristics, PEH have a higher prevalence of substance and alcohol use issues (Fazel et al., 2008). Alongside these health issues, PEH experience loneliness and social isolation (Bower et al., 2018; Sanders & Brown, 2015), and can be the subject of violent crimes (Ellsworth, 2019), particularly amongst people living on the streets (Newburn & Rock, 2006; Sanders & Albanese, 2017) and women (Nilsson et al., 2020).

PEH can experience multiple overlapping issues, or multiple exclusion homelessness (Bramley et al., 2020; Shelton et al., 2012; Tsai et al., 2013). In the United Kingdom, multiple exclusion homelessness has been found to have a gendered dimension, with an estimated 70% of people in England between 2010 and 2014 who experienced homelessness, mental ill‐heath, being a victim of interpersonal violence and abuse, and substance use issues being women (Sosenko et al., 2020). People experiencing multiple exclusion homelessness may find difficulties in attaining adequate housing, due to a lack of co‐ordination and partnership between housing and other services.

In the United Kingdom, where this review is primarily intended to inform policy and practice, the lack of partnership working was thrown into stark contrast during the SARS‐CoV‐2 pandemic beginning in 2019. The response to homelessness during the COVID‐19 pandemic saw closer co‐operation between local government and third sector homelessness organisations; something which the homelessness sector recognised should be the de facto position, rather than a crisis response (Grassian & Boobis, 2021). Alongside a lack of partnership working there are other barriers to services for PEH (Black & Gronda, 2011; O'Carroll & Wainwright, 2019). These include the often‐high levels of bureaucracy involved, the inflexibility of services which includes structural barriers such as needing a contact address or having to travel to services, combined with working practices that actively prevent access to services for certain PEH known as ‘gatekeeping’.

### 1.2 The intervention

At its core, case management is a form of care coordination (Hannigan et al., 2018; Lukersmith et al., 2016). A case manager or team of people assess, plan, and facilitate access to a range of services for a participant (Ponka, 2020). The broad principles of case management are that it is participant driven, pragmatic, flexible, anticipatory, culturally sensitive and offers a single point of contact (Vanderplasschen et al., 2004). Case management often includes practical support, help with the development of independent living skills, acute support during crises, and support for healthcare and contacts in social and professional support systems (De Vet et al., 2013).

To a certain extent, all homelessness services adopt some form of case management as they assess, plan, and coordinate help (Homeless Link, 2019). There are however formalised models of case management structured to fit specific care contexts and the issues faced by people.

### 1.3 Description of the condition

Individuals or households who are currently experiencing, or are at risk of experiencing homelessness.

### 1.4 Description of the intervention

From the literature on case management for PEH (De Vet et al., 2013; Homeless Link, 2019; Munthe‐Kaas, 2018; Ponka, 2020), there are five main models:

- Broker Case Management (BCM)—Case managers assess people and their needs and purchase or coordinate appropriate services. Being mainly used with people facing less complex issues, such as those with mainly housing‐related issues, there is very little service provision by the case worker, who may have a large case load.
- Standard Case Management (SCM)—Similar to the brokerage model in terms of the low intensity of work and the target group, the SCM model is less aligned to the purchase of services for the participant. There is also some level of relationship between case manager and participant, unlike the broker model where this relationship is not important.
- Intensive Case Management (ICM)—The case manager providers a high level of support to the participant to access other service and/or resolve issues of relevance. As ICM involves ongoing comprehensive support, caseloads are kept intentionally small.
- Assertive Community Treatment (ACT)—Rather than a single case manager, ACT draws on a multidisciplinary team or network to support participants within a service.
- Critical Time Intervention (CTI)—Offers time‐limited and structured support during periods of transition, for example moving into permanent accommodation. The aim of CTI is to provide continuity of care during periods of change.

**Figure 1:**
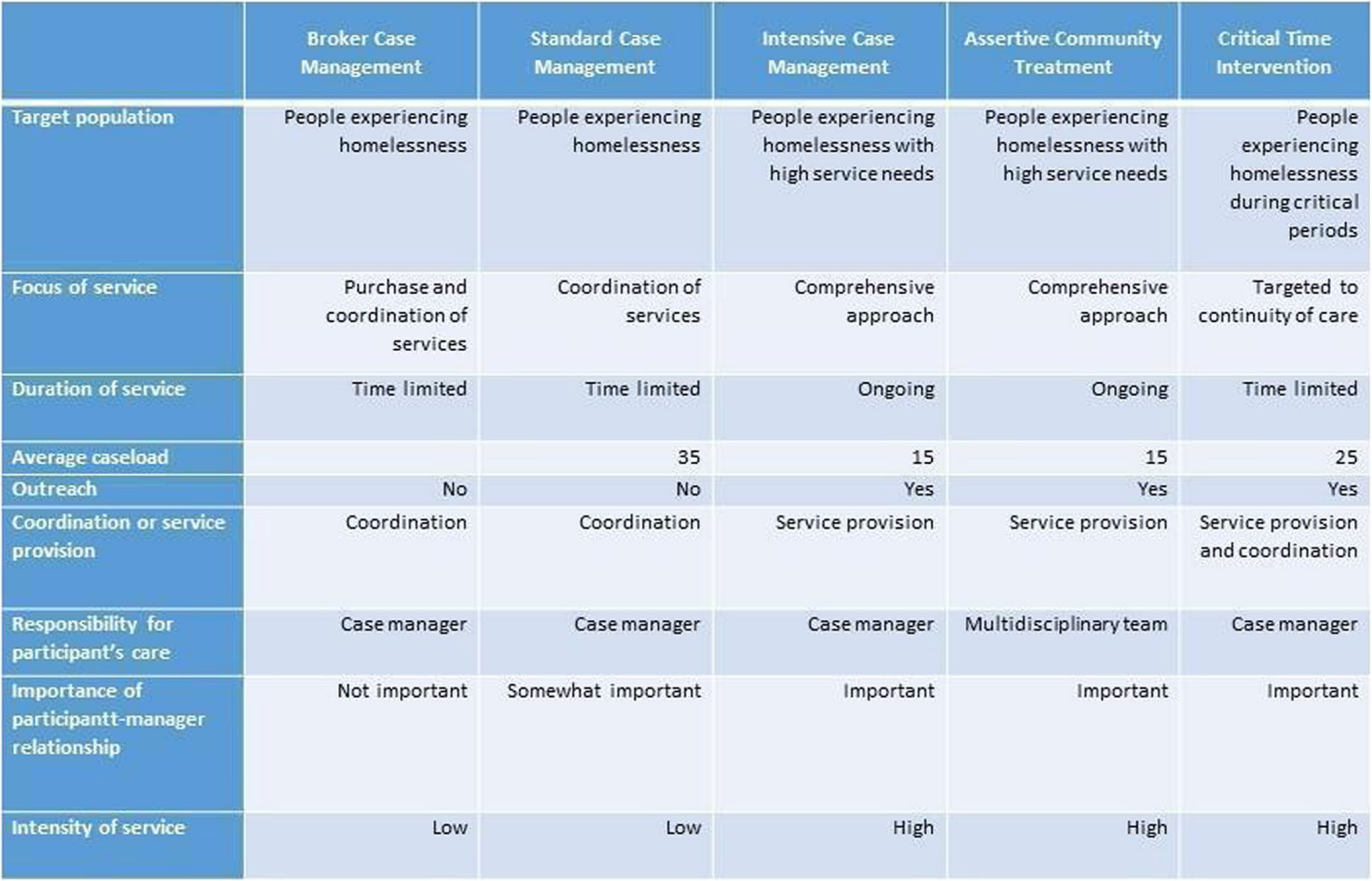
Characteristics of case management models for people experiencing homelessness. *Source*: Adapted from Munthe‐Kaas et al. (2018), Ponka et al. (2020), De Vet et al. (2013), and Homeless Link (2019)

Each of the case management models identified above is structured in distinct ways, summarised in Figure 1. Munthe‐Kaas et al. (2018) describe the different case management models in terms of eight characteristics: (1) focus of services, (2) duration of services, (3) average caseload, (4) whether the service involves outreach, (5) whether the service involves coordination or service provision, (6) who is responsible for the participant's care, (7) the importance of the participant‐case manager relationship, and (8) intensity of service. De Vet et al. (2013) and Ponka et al. (2020) also include the target population when describing the use of case management for PEH.

### 1.5 How the intervention might work

PEH can experience barriers to accessing services. Having a case manager act in a position of authority when interacting with services could potentially overcome or lessen these barriers. However, this view assumes that PEH lack self‐determination (Thomas et al., 2012). Case management can therefore be structured to empower PEH to set and realise their own goals (a strengths‐based approach).

Where PEH experience multiple forms of exclusion and have multiple support issues, this may require them to engage with multiple services, for example, housing and mental health services. However, a lack of co‐ordination amongst services may prevent PEH from receiving the holistic assistance they need in a timely manner—particularly when combined with barriers in accessing services on their own. As a form of service co‐ordination, case management offers a centralised point of contact in referring and brokering access or acting on behalf of the person, and in some models by providing ongoing support with services.

### 1.6 Why it is important to do this review

Systematic reviews and meta‐analyses of case management with PEH have found that this intervention can lead to improvement in people's outcomes (Coldwell & Bender, 2007; De Vet et al., 2013; Munthe‐Kaas, 2018). The most recent published review by Ponka (Ponka 2020) found that standard case management had both limited and short‐term effects on substance use and housing outcomes and showed potential to increase hostility and depression. Intensive case management substantially reduced the number of days spent homeless [standardised mean difference (SMD): −0.22; 95% confidence interval (CI): −0.40 to −0.03], as well as substance and alcohol use. Critical time interventions and assertive community treatment were found to have a protective effect in terms of re‐hospitalisations and a promising effect on housing stability. Assertive community treatment was found to be cost‐effective compared to standard case management.

However, there is only limited evidence of the relative roles of the different types and components of case management in influencing outcomes amongst PEH. Furthermore, PEH are largely treated as a homogenous group in previous reviews, when homelessness can cover a range of different experiences (Amore et al., 2011; Edgar, 2009) and have different causal factors. There are therefore important differences in people's experience of homelessness, for example, along the lines of gender (Bretherton, 2017), that may impact which components of case management are more appropriate and effective with different groups of PEH.

This review proposes to add value to the reviews described above by taking a mixed methods approach, including interventional and observational research. The review team will attempt to disentangle the components of the case management models explored in the research literature, using statistical analysis where feasible. The findings from narrative and any meta‐analytical syntheses will be supported by an analysis of the themes identified from implementation/qualitative research with respect to possible factors that may impact on implementation success. Very few studies focus on a single of these components so such a review may not identify causal effects, but it could still help policymakers to design interventions, and researchers to prioritise parameters that should be tested more rigorously.

## 2 OBJECTIVES

To carry out a mixed methods review to summarise current evidence relating to the components of case‐management interventions for PEH.

We will summarise:

1. What is known about component effectiveness/cost‐effectiveness.
2. Knowledge regarding case management effectiveness, and its components, in relation to the characteristics of the recipients of this intervention.
3. What is known about the implementation and process factors that may impact on intervention delivery in terms of case management approach, intervention components and recipient characteristics.

See Data Extraction and Management for details.

## 3 METHODS

### 3.1 Criteria for considering studies for this review

#### 3.1.1 Types of studies

This is a mixed methods review including both quantitative (effectiveness) and qualitative (implementation) studies. Effectiveness studies will be synthesised with a meta‐analysis where feasible; while a narrative/thematic synthesis will be used to synthesise the factors that may impact on implementation.

##### 3.1.1.1 Quantitative studies

We will include all quantitative study designs where a comparison group is used. This includes randomised controlled trials (RCTs), quasi‐experimental designs, matched comparisons and other study designs that attempt to isolate the impact of the intervention on homelessness using appropriate statistical modelling techniques. These designs are chosen, as the use of a control group helps ensure that changes observed in treatment group participants are due to effects of the intervention, and not attributable to other factors.

As RCTs are accepted as more equipped to infer causality than nonrandomised studies, the potential impact of non‐randomised study designs on effect sizes will be explored as part of the analysis of heterogeneity. Where feasible, for the primary outcomes, sensitivity analyses will be carried out on the basis of study design and risk of bias assessment.

Studies must include an alternative case‐management approach or an inactive comparison condition that could include:

- No treatment.
- Treatment as usual. Details of what this comprises will be extracted.
- Waiting list where service providers or service users are randomly assigned to receive the intervention at a later date. Details of what happens to waitlisted participants will be extracted.
- Attention control, where participants receive some contact from researchers but both participants and researchers are aware that this is not an active intervention.
- Placebo where participants perceive that they are receiving an active intervention but the researchers regard the treatment as inactive.

Studies with no control or comparison group (e.g., pretest/posttest), unmatched controls or national comparisons with no attempt to control for relevant covariates will not be included. Case studies, opinion pieces or editorials will not be included.

##### 3.1.1.2 Qualitative, process and implementation studies

We will include all research designs where data are collected on the views and experiences of service users or providers that have some bearing on factors that may impact on the effectiveness of the case management approach. We will search for data that enables a deeper understanding of why an intervention does (or does not) work as intended, for whom and under what circumstances.

In addition to specific qualitative study designs (such as focus groups and interviews), mixed methods studies, process evaluations, surveys, observational studies (e.g., ethnographic) and secondary data analyses will be included. We will include mixed methods studies where it is possible to extract the data that were collected and analysed using qualitative methods.

If a very large number of studies are identified, the review team will use a ‘Best Fit Approach’ based on a sample of studies using formal qualitative methods, and which are deemed most relevant (see Assessment of Findings).

##### 3.1.1.3 Economic and cost‐effectiveness studies

We will include all research with information on the costs/cost‐effectiveness of interventions and individual components of those interventions.

#### 3.1.2 Types of participants

This review relates to the use of case management with PEH, defined as: (1) people without accommodation, such as those living on the streets, (2) people accessing housing that is either temporary or tied to institutional care, such as hostels, shelters, and other temporary accommodation, or people about to be released from prison without accommodation to return to, (3) people in insecure housing, such as ‘sofa surfers’ or those threatened with violence (Busch‐Geertsema et al., 2016). Studies that include the above groups of PEH will be included irrespective of age, gender, or household type. Studies will include populations from the Global North, given that the social and economic contexts of homelessness are likely to be vastly different to those faced in the Global South (Busch‐Geertsema et al., 2016).

#### 3.1.3 Types of interventions

Interventions that will be included within this systematic review will be those with an explicit description of a case‐management approach whereby a designated case manager supports the homeless person by facilitating integrated access to health and social services and accommodation support.

There are five established case‐management approaches (see The Intervention)

- Broker Case Management (BCM)
- Standard Case Management (SCM)
- Intensive Case Management (ICM)
- Assertive Community Treatment (ACT)
- Critical Time Intervention (CTI)

These specific types of intervention will be included as well as any other interventions that claim to adopt a case‐management approach.

Comparison conditions will include services as usual or an alternative service/intervention.

Inclusion/exclusion criteria for the review are summarised in Supporting Information Appendix 1.

#### 3.1.4 Types of outcome measures

The review will explore a range of housing, health and wellbeing outcomes.

##### Primary outcomes

**Figure 2:**
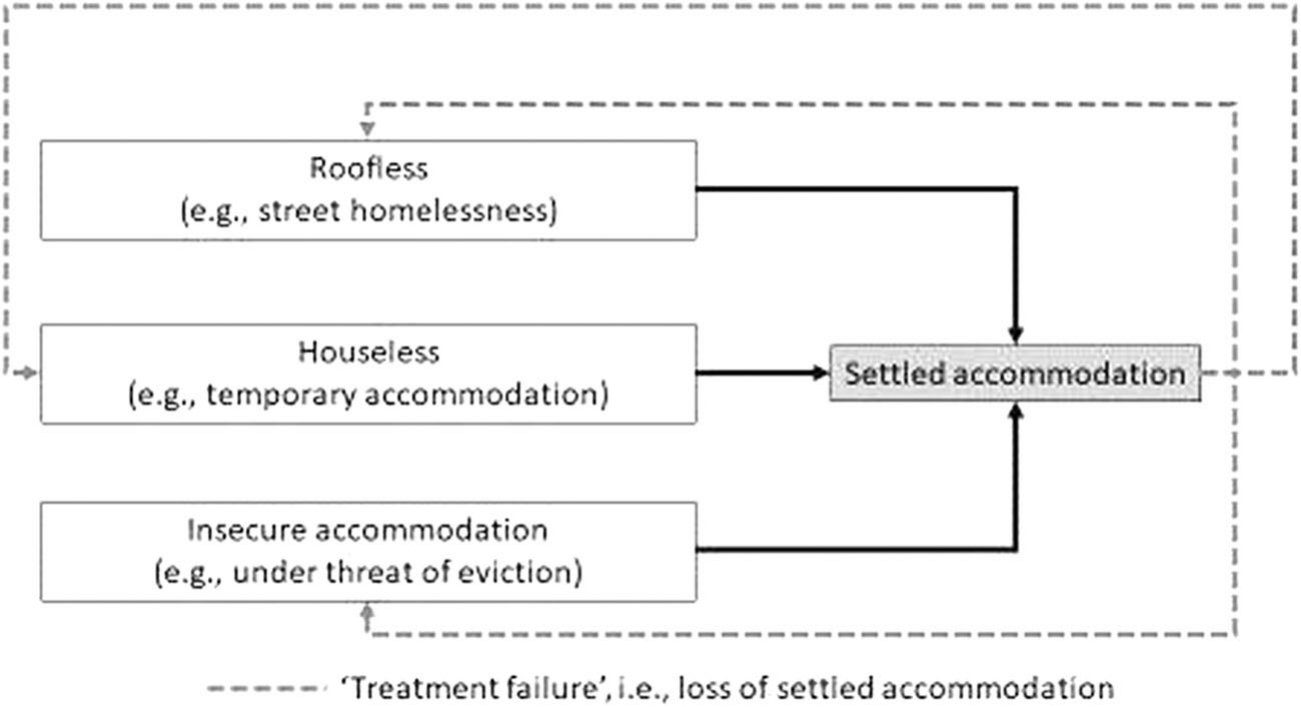
Primary outcome—Settled accommodation

In keeping with Keenan (2020) this review will primarily address how interventions can reduce homelessness and/or increase housing stability. Where case management interventions lead to settled accommodation, for households that lose that settled accommodation and return to *any* state of homelessness, this will be considered ‘treatment failure’ (Figure 2). Likely measures in the reviewed literature may include % returning to the streets or % still in settled accommodation.

Where feasible, the *primary outcomes* will be explored in relation to the characteristics of case management and the individuals receiving it (objectives 1 and 2).

##### Secondary outcomes

Secondary outcomes will include all other outcomes reported by studies which include:

- Access to health and social care services
- Physical health
- Mental health
- Substance use
- Crime/criminalisation
- Employment and income
- Capabilities and wellbeing
- Cost/cost‐effectiveness of intervention

We will also pay attention to implementation and acceptability of interventions and will include a descriptive report of attrition rates or ‘dropout’ from interventions.

### 3.2 Search methods for identification of studies

This systematic review will be based on evidence identified from a specific search for all types of research study published since 1990 exploring case management in homelessness.

This topic‐specific search will supplement the large set of studies already identified from existing evidence and gap maps (EGMs) relating to homelessness (White & Keenan, 2018; White et al., 2018, 2020) and a recent systematic review looking at case‐management in homelessness (Ponka, 2020). The most recent search for intervention studies for the EGM was completed in March 2020 and the most recent search for qualitative and other studies relating to implementation was completed in January 2021. The earliest study identified from these searches was published in 1992.

#### 3.2.1 Electronic searches

Electronic searches will be carried out in 11 databases (Supporting Information Appendix 2).

#### 3.2.2 Searching other resources

To ensure that the search has a very high sensitivity (i.e., it identifies the vast majority of relevant research studies) additional supplementary methods will include reference list follow‐up of included papers and co‐citation tracking based on the *Related records*.^1^ function in Web of Science and the *Related* 2 feature in Microsoft Academic. These are two completely different and complementary approaches to finding additional relevant studies and Microsoft Academic has the additional advantage of including a large body of grey literature within the database. Finally, the web sites explored by White et al. (2018, 2020) for the EGM in March 2020 (see Supporting Information Appendix 2) will also be browsed for any publications in 2020 and 2021. There will be no language restrictions.

It is expected that the majority of included intervention studies will already have been identified within the EGM (White et al., 2018). Additional studies identified will be critically appraised and data extracted for inclusion in the Centre for Homelessness Impact's Homelessness Effectiveness Map: https://centreforhomelessnessimpact.github.io/egm/

### 3.3 Data collection and analysis

#### 3.3.1 Description of methods used in primary research

For evidence on effectiveness, interventions including RCTs and other designs with a comparison group measuring the effectiveness of the case management approach where the comparison group may be usual practice or an alternative intervention.

For evidence on implementation, qualitative and other research gathering views, opinions and experiences of relevance to factors that may impact on the effectiveness of the case management intervention

#### 3.3.2 Criteria for determination of independent findings

It is important to ensure that the effects of an individual intervention are only counted once and the following conventions will therefore apply.

Where the same outcome construct is measured but across multiple time domains, such as through the collection of both posttest and further follow‐up data, the analysis will be conducted and reported separately for different time points. We will split outcome timings into categories of ‘less than a year post‐intervention’ and ‘a year or longer post‐intervention’.

Separate meta‐analyses will be conducted for each outcome and no study will contribute twice to the same forest plot (except where the control group can be split for multi‐arm trials).

#### 3.3.3 Selection of studies

Studies that have not already been included in the EGM as case‐management studies will be screened against the inclusion criteria for eligibility by two independent screeners using EndNote, with recourse to a third reviewer if there are any discrepancies.

#### 3.3.4 Data extraction and management

For all studies, we will undertake dual data extraction, where two authors will both complete data extraction independently for each study. Coding will be carried out by trained researchers. Any discrepancies in screening or coding will be discussed with senior authors until a consensus is reached.

##### 3.3.4.1 Intervention studies

An evidence table has been designed, and piloted, for data extraction of intervention studies based on the coding framework developed by Keenan (2020) (Supporting Information Appendix 3).

We will extract the following data: publication details, intervention details including type of case‐management approach, design and type of research study, population characteristics [including age, gender, household type (individual/family)], any health information, sample sizes, attrition rates, data required for any meta‐analyses, time to follow up, descriptions of the outcomes of interest including instruments used to measure, quality assessment.

Specifically, we will summarise:

1. The type of case management approach (Figure 1), and its components according to the following categories and (preliminary) measures of intensity:

1. Case manager continuity (Named case manager vs. No dedicated case manager)
2. Caseload of the case manager (defined as high ≥21; medium 8–20; light ≤7)
3. Frequency of contact with PEH (defined as very frequent ≥8 times/month; frequent 4–7 times/month; medium 2–3 times/month; occasional ≤once/month)
4. Availability of the support (defined as high 24/7; office hours (guaranteed response) or low <office hours)
5. Level of input PEH have in goal setting and care planning (case manager led or person led)
6. Time‐limit of provision of the support (defined as long term ≥3 years, medium >6 months to < 3 years, short term 3–6 months; very short term <3 months)
7. Location of appointments (institution, community, independent accommodation, remotely)
8. Degree of arranging service provision versus referral/coordinating arrangements to others
9. Team versus individual approach to case management
10. Types of case manager (nonprofessional, with lived experience, professional)
11. Whether there are conditions attached to the support provided (Not conditional vs. conditional)
12. Knowledge regarding case management effectiveness in relation to the characteristics of the recipients of this intervention, which may include:
  a. Type of case management approach
  b. Complexity of needs.
  c. Age.
  d. Household type
  e. Gender.
  f. Type of homelessness experienced
  g. UK national versus non‐UK national
  h. Ethnicity
  i. Care or prison leaver
  j. LCGTQ+
  k. Whether first time or multiple homeless

Additional descriptive information for each of the studies will be extracted and coded to allow for sensitivity and subgroup analysis. This will include information regarding:

- Setting, which type of institutional setting(s) are study participants transitioning from?
- Demographic variables relating to the participants including age, complexity of needs, dependent children, and other relevant population characteristics.

Quantitative data will be extracted to allow for calculation of effect sizes (such as mean change scores (analysed according to the Cochrane handbook section 9.4.5.2) and standard error or pre and post means and standard deviations or binary 2 × 2 tables). Data will be extracted for the intervention and control group on the relevant outcomes measured to assess the intervention effects.

Multi‐arm trials with arms that are not comparing case management against usual care will have just the relevant information extracted. Our table of characteristics will note the un‐included intervention arms.

Where data are available, sensitivity/subgroup analyses will be carried out for the primary outcome (homelessness) and the key secondary outcome (mental health) with regard to the intervention components

##### 3.3.4.2 Implementation studies

An evidence table has been designed and piloted for the data extraction of the implementation (qualitative) studies (Supporting Information Appendix 3). We will extract the following data: publication details, type of case‐management approach, design and type of research study, research question, theoretical approach adopted (if any), setting, participants, recruitment process, method of analysis, themes identified in relation to any of the case management components and recipient characteristics as outlined above, quality assessment.

#### 3.3.5 Assessment of risk of bias in included studies

Where studies have not already been assessed for risk of bias for inclusion in the EGM, assessment of methodological quality and potential for bias will be conducted using the second version of the Cochrane Risk of Bias tool for Randomised controlled trials (Higgins et al., 2020). Non‐randomised studies will be coded using the ROBINS‐ I tool (Sterne et al., 2016). Qualitative, process and implementation studies will be assessed using a tool developed by Campbell. (White & Keenan, 2018). Assessments of risk of bias will be carried out by two reviewers independently with discussion to resolve any differences.

We will not exclude studies based on our assessment of methodological limitations. We will record this information in Summary of Findings Tables to use it to assess our confidence in the review findings.

#### 3.3.6 Measures of treatment effect

It is anticipated that most primary outcomes will be based on binary measures of homelessness and so relative risks will be used to summarise these outcomes. Secondary outcomes reported will likely be based upon continuous variables and so the main effect size metric to be used for the purposes of the meta‐analyses will be the SMD, with its 95% CI.

Within this, Hedges' *g* (Hedges et al., 2010) will be used to correct for any small sample bias as is automatically implemented in the R package ‘meta’.

#### 3.3.7 Unit of analysis issues

If studies involve group‐level allocation, where possible, data will be included that have been adjusted to account for the effects of clustering, typically through the use of multilevel modelling or adjusting estimates using the intra‐cluster correlation coefficient (ICC). Where the effects of clustering have not been taken into account, estimates of effect size will be adjusted following guidance in the Cochrane Handbook. If ICC is not reported external estimates will be obtained from studies that provide the best match on outcome measures and types of clusters from existing databases of ICCs (Ukoumunne et al., 1999) or other similar studies within the review.

#### 3.3.8 Dealing with missing data

If study reports do not contain sufficient data to allow calculation of effect size estimates authors will be contacted to obtain necessary summary data, such as means and standard deviations or standard errors. If this route is not successful we will employ standard methods to calculated a standardised mean difference from reported statistics or graphics in the paper (Rosnow & Rosenthal, 1996; Rosnow et al., 2000). We may also use an online calculator to facilitate this (Lipsey & Wilson, 2001). If no information is forthcoming the study cannot be included in meta‐analysis and will instead be included in a narrative synthesis.

#### 3.3.9 Assessment of heterogeneity

Heterogeneity will be assessed through visual inspection of the forest plot and checking for overlap of confidence intervals and second through the *I* ^2^ and *τ* ^2^ statistics.

#### 3.3.10 Assessment of reporting biases

If sufficient numbers of studies are included (at least 10) a funnel plot and Egger's linear regression test will be included to check for publication bias across included studies (Sterne & Egger, 2006).

To ensure robustness of the review and to account for individual studies that appear to exert an undue influence on findings, process sensitivity analysis will also be carried out on domains relating to the quality of the included studies.

#### 3.3.11 Data synthesis

All statistical analyses will be conducted using the R program using the ‘meta’ library (Balduzzi et al., 2019). A random‐effects analysis (REM) is chosen as the hierarchical linear model. This decision to employ a REM is made for two reasons. First, we expect studies to vary substantially in terms of population served, training of case managers, outcomes assessed, and study designs. Second, under the random‐effects model the weights assigned to each individual study are more reasonable as it considers that the effect observed within each study are based on a sample from a population with an unknown mean.

Meta‐analysis will be conducted to test effectiveness of interventions to improve case‐management approaches across various domains relating to homelessness. The outcomes related to homelessness are both binary and continuous and so the effect size metrics chosen will be relative risks and standardised mean differences.

#### 3.3.12 Subgroup analysis and investigation of heterogeneity

We will conduct a number of subgroup analyses, where sufficient data are available, to explore whether study, intervention or sample characteristics influenced the overall effect of the intervention on each outcome. The moderating variables include:

- The methodological quality of the study (study design/risk of bias assessment),
- The age of participants,
- The gender of participants,
- The ethnicity of participants
- Type of homelessness (according to the FEANTSA classification; FEANTSA, 2017),
- Whether the intervention was aimed at single people or families,
- Setting of the intervention
- How the intervention was classified (according to the framework discussed earlier) as aiming to increase access to services through improving the availability, acceptability or affordability of the programme,
- The intervention components (see Analysis of Finding)

We are particularly interested in teasing apart the contributions of different intervention components to outcomes. Where sufficient studies are identified (at least 10) we will include intervention component information (either continuously or categorically measured) for the intervention components listed in the Analysis of Findings in a meta‐regression. Bubble plots and regression coefficients and their 95% confidence intervals will summarise the results.

#### 3.3.13 Sensitivity analysis

Where feasible, for the primary outcomes, sensitivity analyses will be carried out on the basis of study design and risk of bias assessment.

#### 3.3.14 Summary of findings and assessment of the certainty of the evidence

##### Treatment of qualitative research

We will describe the characteristics of included studies in terms of the methods used to capture data on the factors that may impact on intervention implementation and success; the number of interviews/focus groups/observations that have taken place, who participated and the nature of qualitative data collection (type and time taken).

The categories included in the EGM describe the factors that impact upon interventions and the implementation of these across the gathered studies. These categories were developed using an iterative process and were initially based on the implementation science framework (Aarons et al., 2011). The categories were then independently piloted against process evaluations and agreement was reached by researchers in the Centre for Homelessness Impact, the Campbell Collaboration, Campbell UK and Ireland, and Herriot‐Watt University. The five broad categories or levels of influence agreed are contextual factors, policy makers/funders, programme managers/implementing agency, staff/case workers and recipients. These factors will be considered by the review team but the synthesis will be driven by the evidence gathered and new themes incorporated as appropriate.

As with Keenan et al., 2020 framework synthesis is the approach that will be adopted, supported by the use of NVivo or Excel.

This Framework synthesis will comprise five methodological stages:

1. Familiarisation—with issues and ideas around the topic by an initial screening of relevant studies identified in the search
2. Framework Selection—to agree the conceptual framework or logic model to provide a potential set of themes or concepts that may affect implementation success
3. Indexing—to data extract information from each study in relation to their main characteristics and findings
4. Charting—to group the study findings in relation to the themes in the Framework and any new themes/sub‐themes derived directly from the inductive data‐driven process.
5. Mapping and Interpretation—the derived themes will be considered in light of the interventional research and its components

These stages are often overlapping and may be revisited throughout the process.

At the charting stage, in the event of a very large amount of relevant evidence, purposive sampling (Booth et al., 2016) will be employed to include research spanning geography, targeted populations and types of intervention to exhibit an accurate representation of the case management programmes available with the prioritisation of high quality studies (as assessed by critical appraisal). The selected process evaluations should present the most ‘rich’ and ‘thick’ data (Booth et al., 2016) from the studies included.

##### 3.3.14.1 Reflexivity

Review author reflexivity, that is, the potential for pre‐existing views to influence review conclusions will be considered at all stages of the review. We will explore any author or subject expert views and positions that could influence the review's conclusions and ensure that steps are taken to minimise any potential for bias.

##### 3.3.14.2 Synthesis of findings

The overall synthesis will be guided by the method proposed by Harden (2018) for integrating contextual features from the qualitative research with findings from the effectiveness studies.

At the final mapping and interpretation stage, the team will collaborate closely with CHI as well as the panel of experts they convened who will consider these themes in light of the available empirical literature. For the interventions available for meta‐analysis, implementation evidence directly linked to these interventions, and any evidence in relation to component interventions, will be considered in light of specific adjustments to the interventions that might be considered.

At this stage, based on any evidence available, there will also be discussion on the most cost‐effective level of support (level of each component) for each population. Overall, the aim of this synthesis will be to help policy makers design interventions, and researchers to prioritise parameters that should be tested more rigorously.

Once we have finished preparing the review findings, we will examine each finding, identify factors that could influence the implementation of the intervention/s, and develop prompts for future implementers. These prompts will be presented in the implications for practice section. They are not intended to be recommendations but will be phrased as questions to help implementers consider the review findings within their context.

## CONTRIBUTIONS OF AUTHORS

- Content: Ian Thomas, Ben Hannigan, Robin J. Smith
- Systematic review methods: Alison L. Weightman, Mala Mann, Simone Willis, Rhiannon Cordiner
- Statistical analysis: Mark Kelson
- Information retrieval: Mala Mann, Lydia Searchfield

## DECLARATIONS OF INTEREST

None to declare.

## PRELIMINARY TIMEFRAME

Approximate date for submission of the systematic review. December 2021.

## PLANS FOR UPDATING THIS REVIEW

We will update this review if a significant new body of research is available and funding is secured.

## SOURCES OF SUPPORT

**Internal sources**

- New Source of support, UK
  No internal sources of support
**External sources**
- Centre for Homelessness Impact, UK
https://www.homelessnessimpact.org/

## Supporting information

Supporting information.

## Footnotes

^1^
Related records are those that cite at least one document in their reference list that was cited by the parent (seed) document. The list is ranked by the number of shared references and anecdotal evidence from SURE suggests that documents with three or more shared references are closely related to the topic area of the seed paper.

^2^
Related articles are those where the title and abstract look similar to those of the seed paper based on an algorithm which develops a similarity score based on ‘academic concepts’ (the semantic meaning of the text).
