# Supplementary material for: PROTOCOL: Exploring the effect of case management in homelessness per components: A systematic review of effectiveness and implementation, with meta‐analysis and thematic synthesis
Source: Campbell Syst Rev. 2022 Feb 23;18(1):e1220. doi: 10.1002/cl2.1220 (PMC8866910; doi:10.1002/cl2.1220)
Supplement: Supplementary file 1 — Supporting information. [file CL2-18-e1220-s001.docx]

# Appendices

## 1 Appendix 1:  Inclusion/Exclusion Summary

|  | **Inclusion criteria** | **Exclusion criteria** |
| --- | --- | --- |
| **Intervention** | Case-management, whereby the case-manager supports the person or household experiencing, or at risk of experiencing, homelessness by facilitating integrated access to health & social services and accommodation support | Case-management where housing support is not part of the intervention |
| **Study type: Quantitative** | Quantitative study designs with a comparison group    Research with data on the costs/cost-effectiveness of interventions and individual components of those interventions. | Designs without a comparison group (eg pre/post-test) or where the comparison group is from another study |
| **Study type: Qualitative** | Qualitative study designs and process evaluations where data are collected on views and experiences of participants or case-managers |  |
| **Population** | Individuals or households who are currently experiencing, or at risk of experiencing, homelessness as defined by the ETHOS typology (FEANTSA 2017) | Populations from countries other than high-income countries (as defined by the World Bank 2020)    Populations living in housing defined as inadequate but not as experiencing, or at risk of experiencing, homelessness. |

## 2 Appendix 2:  Search Strategy

This case-management specific search strategy supplements the findings from the Campbell EGM.  Example strategies are provided below for Scopus and Medline (Ovid).  These searches will be tested for their ability to identify known relevant studies with good sensitivity before adapting the search to the other databases.

In all, the following databases will be searched:  ASSIA [ProQuest], CINAHL [EBSCO], Cochrane Library, ERIC [EBSCO], International Bibliography of the Social Sciences (IBSS) [ProQuest], Medline [OVID], NHSEED, PsycINFO [OVID], Scopus [Elsevier], Web of Science (Science Citation Index & Social Science Citation Index) [Clarivate].

In addition the web sites explored by White et al (2020) to December 2019 will be browsed for any publications in 2020 and 2021:

Homeless Hub https://www.homelesshub.ca/

European observatory on homelessness https://www.feantsaresearch.org/en/publications

United State interagency council on homelessness http://www.usich.gov/

ETHOS http://ethos.bl.uk/Home.do

WHO ICTRP http://apps.who.int/trialsearch/

Focus on Prevention http://www.preventionfocus.net/

Social Policy and Practice http://www.spandp.net/

FEANTSA https://www.feantsa.org/

National Coalition Homeless https://nationalhomeless.org/

Homelessness Australia https://www.homelessnessaustralia.org.au/

Mission Australia https://www.missionaustralia.com.au/publications/position‐statements/homelessness

National Alliance to end homelessness https://endhomelessness.org/

Institute of global homelessness https://www.ighomelessness.org/

Homelessness link https://www.homeless.org.uk/

Crisis https://www.crisis.org.uk/about‐us/how‐we‐work/

Housing first https://housingfirsteurope.eu/about‐the‐hub/

Canadian Alliance to end homelessness https://housingfirsteurope.eu/about‐the‐hub/

Social work and policy institutes http://www.socialworkpolicy.org/research/homelessness.html

Association of housing advice services https://www.ahas.org.uk/

Centre point https://centrepoint.org.uk/

Homelessness trust funds https://housingtrustfundproject.org/htf‐elements/homeless‐trust‐funds/

Meliville charitable trust https://melvilletrust.org/category/resources‐reports/

Conrad H Hilton foundation https://www.hiltonfoundation.org/priorities/homelessness#resources

Abt Associates https://www.abtassociates.com/

Mathematica https://www.mathematica‐mpr.com/

American Institutes of Research https://www.air.org/

Rand https://www.rand.org/

MDRC https://www.mdrc.org/

**Scopus**

## *( ( ( TITLE-ABS-KEY ( (*evict**OR* homeless**OR* "housing excl*"*OR* "residential stability"*) ) )  OR  ( TITLE-ABS-KEY ( ( (*street**OR* private*OR* improvised*OR* shelter**OR* emergency*OR* temporar**OR* insecure*OR* overcrowded*OR* precarious*OR* stable*OR* marginal**)* near/3*(*dwell**OR* hous**OR* home**OR* accommodat**) ) ) )  OR  ( TITLE-ABS-KEY ( (*street*AND*near/3*(*life*OR* living*OR* lives*OR* youth**OR* child**OR* people*OR* person**) ) ) )  OR  ( TITLE-ABS-KEY ( (*runaway**OR* "Run away from home"*OR* "Running away"*OR* "Ran away"*OR* "Going missing"*OR* "Bag lady"*OR* houseless**OR* unhoused*OR* "without a roof"*OR* roofless*OR  (*rough*AND*near*AND*sleep**)  OR* destitut**OR* "Skid row*"*OR* "sleepers out"*) ) )  OR* (*TITLE-ABS-KEY* ((based or housed or residen* or temporar*) NEAR shelter?) OR (*TITLE-ABS-KEY* (temporar* NEAR (accommodat* or home? or hous*)) OR*( TITLE-ABS-KEY ( (*"Pathways to Housing"*OR* "Homeless Veterans Reintegration Program"*OR* "access program*"*OR* "Supported Housing"*OR* "Housing Program"*OR* "HUD-VASH"*) ) )  OR  ( TITLE-ABS-KEY ( (*"Sober Transitional Housing and Employment Project"*OR* "sober house placement*"*OR* "Housing ladders"*OR* "Staircase housing"*OR* "low threshold housing"*OR* "housing status"*) ) )  OR  ( TITLE-ABS-KEY ( ( (*based*OR* housed*OR* residen**OR* temporar**)* near/2*AND*shelter**) ) ) )  AND  ( TITLE-ABS-KEY ( (*"case manage*"*OR* "assertive community treatment*"*OR* "critical time intervention*"*OR* "care manage*"*OR* "coordinated care"*OR* "managed care"*OR* "care coordination"*) ) ) )  OR  ( TITLE-ABS-KEY ( (*"Housing first"*OR  (*at*AND*home/chez*AND*soi*)  OR* "homebase community prevention program*"*OR* "mckinney program*"*) ) )*

**Medline (Ovid)**

Database(s): **Ovid MEDLINE(R)**
Search Strategy:

| **#** | **Searches** |
| --- | --- |
| 1 | homeless persons/ or homeless youth/ or runaway behaviour/ |
| 2 | (evict* or homeless* or "housing excl*" or "residential stability" or ((street* or private or improvised or shelter* or emergency or temporar* or insecure or overcrowded or precarious or stable or marginal*) adj3 (dwell* or hous* or home* or accommodat*)) or (street adj3 (life or living or lives or youth* or child* or people or person*)) or runaway* or "Run away from home" or "Running away" or "Ran away" or "Going missing" or "Bag lady" or Houseless* or Unhoused or "without a roof" or Roofless or (rough adj3 sleep*) or Destitut* or "Skid row*" or "sleepers out").ti,ab,kw OR ((based or housed or residen* or temporar*) adj2 shelter?).tw.kw. OR (temporar* adj2 (accommodat* or home? or hous*)).tw,kw |
| 3 | ("Pathways to housing" or "homeless veterans reintegration program" or "access program*" or "supported housing" or " housing program*" or "HUD-VASH").ti,ab,kw. |
| 4 | ("Sober transitional housing and employment project" or "sober house placement" or "housing ladders" or "staircase housing" or "low threshold housing" or "housing status").ti,ab,kw. |
| 5 | ((based or housed or residen* or temporar*) adj2 shelter*).ti,ab,kw. |
| 6 | or/1-5 |
| 7 | case management/ |
| 8 | ("case manage*" or "assertive community treatment*" or "critical time intervention*" or "care manage*" or "care coordination" or "managed care").ti,ab,kw. |
| 9 | 7 or 8 |
| 10 | 6 and 9 |
| 11 | ("housing first" or "at home/chez soi" or "homebase community prevention program" or "mckinney program").ti,ab,kw. |
| 12 | 10 or 11 |

## 3 Appendix 3:  Summary of Findings Tables

**Evidence Table:  Interventions**

| First author & year | Study design | Type of CM intervention (include also: team or individual, type of case manager, arrangement/referral) | Intervention components  (include: Continuity, caseload,  frequency of contact, availability, time limit, any conditionality) | Theory of change | Location of appointments  (Institution, community, independent accommodation; remote or in-person?) | Participants  (include: age, gender, ethnicity, household type, complexity of needs.) | Homelessness status[[1]](#_ftn1)  (FEANTSA 2017; First time or previous experience) | Health status: Phys/Ment/ Substance use | Sample size | Follow-up period(s) Attrition | Outcomes | Limitations | Funding source(s)/potential CoI | Notes |
| --- | --- | --- | --- | --- | --- | --- | --- | --- | --- | --- | --- | --- | --- | --- |
| Study A |  |  |  |  |  |  |  |  |  |  |  |  |  |  |
| Study B |  |  |  |  |  |  |  |  |  |  |  |  |  |  |

**Notes**

- Team versus individual approach to case management
- Types of case manager (non-professional, with lived experience, professional etc.)
- Degree of arranging service provision versus referral/coordination arrangements to others
- Continuity (Named case manager vs No dedicated case manager)
- Caseload (defined as high ≥21; medium 8-20; light ≤7)
- Frequency of contact (defined as very frequent ≥8 times/month; frequent 4-7 times/month; medium 2-3times/month; occasional ≤once/month)
- Availability of the support (defined as high 24/7; office hours (guaranteed response) or low <office hours)
- Adoption of a strengths-based, a trauma-informed, asset focussed, harm reduction or other approach
- Time-limit of provision of the support (defined as long term ≥3 years, medium >6 months to < 3 years, short term 3-6 months; very short term <3 months)
- Conditionality of the support provided:  Not conditional versus conditional on attendance rate

**Evidence Table – Implementation**

| First author & year | Study design | Research question | Research theory | Setting | Participants | Recruitment process | Method and process of analysis | Themes | Limitations | Funding source(s) | Notes |
| --- | --- | --- | --- | --- | --- | --- | --- | --- | --- | --- | --- |
| Study A |  |  |  |  |  |  |  |  |  |  |  |
| Study B |  |  |  |  |  |  |  |  |  |  |  |

[[1]](#_ftnref1) See ETHOS Light https://www.feantsa.org/download/fea-002-18-update-ethos-light-0032417441788687419154.pdf
